# The application of Phase 0 and microtracer approaches in early clinical development: past, present, and future

**DOI:** 10.3389/fphar.2024.1369079

**Published:** 2024-03-18

**Authors:** A. F. Roffel, E.-J. van Hoogdalem

**Affiliations:** ICON plc, Groningen, Netherlands

**Keywords:** Phase 0, microdosing, drug discovery and development, exploratory clinical trials, GMP

## Abstract

Phase 0 microdosing studies were introduced to the drug development community approximately 20 years ago. A microdose is defined as less than 1/100th of the dose calculated based on animal data to yield a pharmacological effect in humans, with a maximum of 100 μg, or 30 nmoles for protein products. In our experience, Phase 0 microdose studies have not been fully embraced by the pharmaceutical industry. This notion is based on the number of Phase 0 studies that we have been involved in. Thus, we conducted at least 17 Phase 0 microdose studies in the Zero’s (on average, two per year), but in the years beyond this, it was only 15 studies (1.4 per year); in these latter years, we did conduct a total of 23 studies which employed an intravenous (i.v.) microdose for absolute bioavailability (ABA) assessments (two per year on average), which are the most used and potentially informative type of clinical study using a microdose, albeit they are formally not microdose studies. In the current review, we summarize the past use of and experience with Phase 0 microdose designs in early clinical development, including intravenous ^14^C microdose ABA studies, and assess what is needed to increase the adoption of useful applications of Phase 0/microdose studies in the near future.

## 1 Introduction

Phase 0 microdosing studies were introduced to the drug development community approximately 20 years ago (Lappin and Garner, 2003; European Medicines Agency (EMA) position paper on non-clinical safety studies to support clinical trials with a single microdose, EMA, 2003; FDA Guidance on Exploratory IND Studies, 2006; EMA concept paper on the development of a Committee for Medicinal Products for Human Use (CHMP) guideline on the non-clinical requirements to support early Phase I clinical trials with pharmaceutical compounds, EMA, 2006). A microdose is defined as less than 1/100th of the dose calculated based on animal data to yield a pharmacological effect in humans, with a maximum of 100 μg, or 30 nmoles for protein products.

The rationale for developing and allowing Phase 0 microdose studies is summarized in Table 1. As an overall goal, quicker and more efficient drug development and approval, especially through de-selecting products that are unlikely to succeed, were sought.

**TABLE 1:** Rationale for the concept of Phase 0 microdose studies.

1. To assess early in clinical development the physiologic, pharmacokinetic (PK) and/or pharmacodynamic properties of an investigational drug in humans, including binding to a target using imaging techniques, based on limited non-clinical data compared to conventional first-in-human studies that escalate into pharmacologically active dose levels
2. To support go/no-go decisions based on data in humans, which conceptually trump animal data
3. To assess pharmacokinetics in humans, especially in those cases in which the animal pharmacokinetics does not readily allow for human pharmacokinetics predictions, or to compare two or more related drug candidates and select the most promising molecule for continuous clinical development

In its 2006 Guidance, the Food and Drug Administration (FDA) stated that sponsors were not taking full advantage of the allowed flexibility in the amount of data that need to be submitted with an investigational new drug (IND) application, such to be based on the goals of the proposed investigation, the level of testing in humans, and the expected risks. The guidance was written to indicate what level of chemistry, manufacturing, and controls (CMC), and non-clinical data would be expected and what clinical approaches could be considered when planning early, exploratory studies in humans.

Phase 0 (or exploratory IND studies) was defined as follows:

- conducted very early in Phase 1;
- involving very limited human exposure, i.e., limited numbers of subjects (healthy volunteers or patients), limited dose range (mostly sub-pharmacological or pharmacologically active but not toxic), and a limited period of time (up to 7 days of dosing);
- having no therapeutic or diagnostic intent.

**FIGURE 1:**
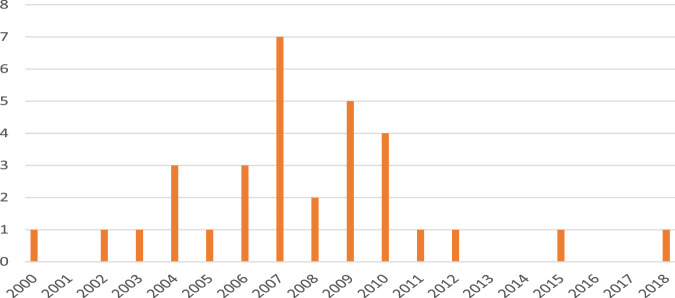
Number of Phase 0 studies conducted in our institute by calendar year, 2000–2018.

In our experience, Phase 0 microdose studies have not been fully embraced by the pharmaceutical industry. This notion is based on the number of Phase 0 studies that we have been involved in (as shown in Figure 1). Thus, between 2000 and 2007, we conducted at least 17 Phase 0 microdose studies (on average, two per year), but in the years 2008–2018, only 15 Phase 0 studies were conducted (1.4 per year); in these latter years, we did conduct a total of 23 studies which employed an intravenous (i.v.) microdose for absolute bioavailability (ABA) assessments (two per year on an average), which are the most used and potentially informative type of clinical study using a microdose, albeit they are formally not microdose studies (see further below).

**FIGURE 2:**
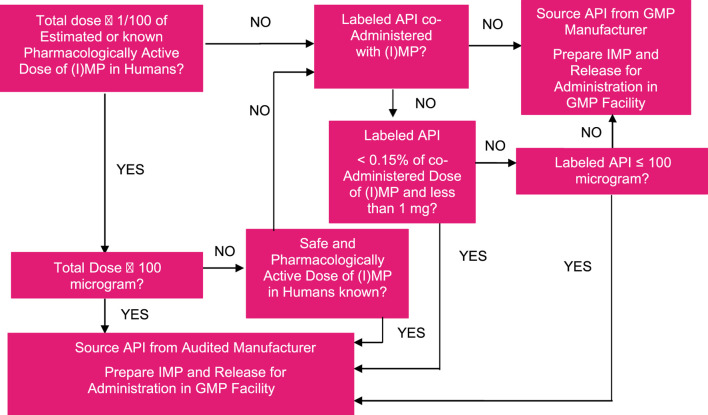
Example of a decision tree for assessing the acceptable manufacturing quality of radiolabeled or stable isotope-labeled product for use in Phase 0 microdose or microtracer studies.

One could wonder why Phase 0 microdose studies are not being employed more often. As common criticism obtained from potential users, we often hear the following objections: (1) the uncertain scalability of pharmacokinetics (PK) for oral drugs from a microdose to a pharmacological dose, especially an issue in the case of dose-dependent absorption or a saturable transporter or enzyme systems being involved in the disposition of the drug; (2) particularly in the first 10–15 years, less so today, the need to work with a ^14^C-labeled drug (in today’s world, LC-MS/MS may well be an option); (3) limited worldwide capacity for accelerator mass spectrometry (AMS) needed for the quantification of low ^14^C plasma concentrations; (4) the need to have a good manufacturing practice (GMP)-quality ^14^C-labeled drug for Phase 0 microdose studies as per company requirements, even though the field (including regulators) will allow the non-GMP material under certain predefined, risk-based circumstances (see Figure 2 for a potential decision tree); (5) the need to do full first-in-human (FIH) in case one decides to proceed with development based on the outcome of the Phase 0 study (which decision is based on human plus non-clinical data rather than just non-clinical data in such case, i.e., a better-informed decision).

As a result, Phase 0 microdose studies have not been fully embraced by many companies and researchers. In the current review, we summarize the past use of and experience with Phase 0 microdose designs in early clinical development, including i.v. ^14^C microdose ABA studies, and assess what is needed to increase the adoption of the informative applications of Phase 0/microdose studies in the near future.

## 2 Bioanalysis (AMS)

The quantification of drug concentrations in plasma from Phase 0 microdose PK studies is most often done by introducing ^14^C labeling into the drug molecule. Specifically, because small mass doses of drugs are being tested, the amount of ^14^C in the drug dose is generally very low, in the order of 3.7–37 kBq (100–1,000 nCi). This often necessitates the use of AMS to accurately quantify drug concentrations, which is perceived by some as a reason not to favor Phase 0 microdose PK studies since worldwide capacity is limited and costs are perceived as prohibitory. In this respect, it should be mentioned that AMS providers have been known to increase capacity over recent years and that some sponsors have decided to set up in-house AMS capacity in order to be able to support Phase 0/microdose/microtracer approaches internally.

In selected cases, ultrasensitive LC-MS/MS can be used for the quantification of drug concentrations in plasma from Phase 0 microdose studies. This negates the need for ^14^C-labeled drug material and bioanalysis using AMS.

## 3 Dosimetry, GMP quality

The low amounts of ^14^C, as commonly used in an investigational drug dose in a Phase 0 microdose PK study, are generally exempted from the requirement to submit human dosimetry calculations. As far as we know, the exemption limit is 1,000 nCi (or 1 µCi) in the United Kingdom and the United States (FDA Guidance on Mass Balance Studies, 2022; equivalent to 37 kBq) and 0.1 MBq (2.7 µCi) in the Netherlands. These lenient requirements are regarded as acceptable based on the notion that the very small amount of ^14^C in up to 100 µg of a small molecule drug will not lead to a radiation burden for a human being above Category I of the International Commission on Radiological Protection (ICRP) (ICRP, 1991), which is associated with trivial risk of health damage (less than 1 in 1 million), generating increased knowledge, and at least a minor societal benefit.

Moreover, based on a risk analysis, several parties in the field (including not only our company but also several sponsors) allow the use of well-characterized but non-GMP ^14^C drug [active pharmaceutical ingredient (API)] in human microdose studies; any non-labeled material in the formulation should be produced under GMP, and this also applies to the manufacture of the investigational medicinal product/drug product. Quality control of the formulation is required and advised as such since issues with adsorption may occur with increased likelihood due to the low dose and low amount of ^14^C used. This level of leniency in applying GMP principles to microdoses is based on considerations around the allowed quantities of (uncharacterized) impurities in the drug material for early clinical investigations and their expected lack of safety or quality complications, as explained in ICH Q7 (2000), ICH Q3A R2 (2006), and FDA’s guidance for industry (2008). An example of a decision tree to decide on the acceptable manufacturing quality of radiolabeled or stable isotope-labeled drug product for use in the Phase 0 microdose or microtracer studies is shown in Figure 2.

## 4 Regulatory environment

The validation of the concept of Phase 0 microdose studies happened in the Zero’s partially also through two European Research Consortia (Lappin et al., 2006; Lappin et al., 2010; Lappin et al., 2011); this included building up an understanding about the necessary interactions with, and understanding on the part of, Regulatory Authorities. As a result, the regulatory adoption of the concept of Phase 0 microdose studies, mainly if not exclusively PK studies, has been without major hurdles in our experience, and this remains the case today (European Clinical Trial Regulation, EU CTR, 2014). As a default, it is advised to adhere to the requirements as laid down in International Council for Harmonization (ICH) M3 R2 (2009); as additional non-clinical data, Regulatory Authorities may well appreciate having *in vitro* genotoxicity testing and hERG inhibition data available as well. This has, in large part, not been an issue since many, if not all, sponsors will run these specific non-clinical tests before entering into clinical studies, be it Phase 0 or Phase 1.

## 5 Applications of Phase 0/microdose studies and related designs

### 5.1 Microdose PK studies

Most, if not all, microdose studies that are being conducted, or have been evaluated for potential conduct, in healthy volunteers were microdose PK studies, aimed at assessing the PK properties of one or more novel candidate drugs, including a preliminary assessment of their metabolic fate. The objective of such studies is to decide whether a drug candidate shows PK properties in humans, including metabolic stability, that makes it suitable for clinical development, or to select the best possible candidate from multiple related molecules. In addition to microdose PK candidate characterization or selection studies, microtracer doses of ^14^C are also being employed in i.v. microdose ABA studies (discussed further below) and microtracer ADME studies. See Table 2 for an overview of the frequency of the various designs used in early clinical PK studies in our institute.

**TABLE 2:** Frequency of the various designs used in early clinical PK studies with a radiolabeled or unlabeled drug (selected Phase 0 studies only) in our institute over a recent 12-year timeframe.

Phase 0 microdose PK with one drug candidate	8
Phase 0 microdose PK with multiple drug candidates	7
Microtracer ^14^C ADME	10
Combined regular dose ADME with microdose ABA	9
Combined microtracer ^14^C ADME with microdose ABA	7
Stand-alone microdose ABA	6
Oral and i.v. ^14^C within FIH	1
Regular dose ^14^C ADME (for references)	76

### 5.2 Design

The design of such studies is often straightforward and will consist of single-dose administration (up to 100 µg and up to 1/100th of the human pharmacologically active dose), followed by up to 168 h of blood sampling for the plasma concentration measurements of total radioactivity (if using ^14^C labeling) and (^14^C-labeled) parent drug. Depending on the available non-clinical data and research questions, the microdose may be administered both orally and intravenously even for an oral drug. Such a design is especially informative in case one needs to obtain an insight into systemic plasma clearance (CL), volume of distribution (Vd), ABA, or the extent of metabolism. Study subjects are most often healthy volunteers (men, or men and women—those of childbearing potential allowed), and the cohort size can be as small as 4 but is more often 6. When comparing multiple drug candidates, a cross-over design within one cohort may be considered, as per the ICH M3 R2 (2009). Placebo control is uncommon since no safety observations are expected; however, placebo treatment may be useful when assessing pharmacodynamic (PD) parameters, such as allowed under approaches 3, 4, and 5, as defined in ICH M3 R2 (2009). The urinary excretion of drug or total radioactivity may be assessed if considered informative.

### 5.3 Non-clinical package

The non-clinical package supporting an early microdose PK study in humans in daily practice almost always consists of the data, as defined per ICH M3 R2 (2009), even though the guidance clearly states that these are examples and that other approaches could be considered. With regard to the required extended single-dose toxicity study with toxicokinetic data, an attractive approach has been to only generate data from i.v. administration (at a conservative dose level of 1000-fold the planned human dose to be administered orally or intravenously); in some regions, Regulatory Authorities have been known to request mutagenicity testing and data on hERG inhibition as well.

### 5.4 Early candidate selection studies

As an early (2006) example of a Phase 0 microdose PK candidate selection study, after the completion of the Consortium for Resourcing and Evaluating AMS Microdosing (CREAM) and European Union Microdose AMS Partnership Programme (EUMAPP) consortia to further establish the concepts of such studies (Lappin et al., 2006; Lappin et al., 2010; Lappin et al., 2011), we conducted a trial for an oral histamine H1 receptor antagonist program in insomnia, where having good estimates of human PK and information about the shape of the concentration–time curve was critical for compound selection. Preferably, the drug was to show a rapid onset and short duration of action, and exhibit low variability. The study generated microdose PK data for four novel compounds, labeled with ^14^C, all derivatives of R-dimethindene, plus diphenhydramine as the reference compound (being an over-the-counter antihistamine used off-label for sleep induction), with oral as well as i.v. dosing. The data were used for advancing the compound with the most favorable PK properties (Madan et al., 2009). Interestingly, one of the investigational compounds, defined as NBI-1, had already been tested in humans and, therefore, also served to add confidence to the PK data for the investigational compounds at the sub-therapeutic dose. PK data for diphenhydramine and NBI-1 suggested that there was good scaling between the microdose and pharmacological dose, and the data obtained for the four investigational molecules allowed them to be ranked based on oral bioavailability, elimination half-life, and PK variability, which led to the selection of NBI-2 as the most promising drug candidate, with the highest bioavailability and short plasma elimination half-life. The study employed separate cohorts of healthy males aged 18–55 years for each compound, with cross-over comparison of oral and i.v. (10-min infusion at 0.4 mL/min) dosing and 7 days (168 h) wash-out between treatments within each cohort. Both oral and i.v. treatment comprised 100 µg of drugs with 7.4 kBq (200 nCi) of ^14^C; PK sampling was done for 48 h after each dose, which turned out to be appropriate, given the observed plasma elimination half-lives of 7–15 h of the parent drug across the compounds tested.

As another successful example, Phase 0 ^14^C microdosing studies were conducted starting from 2004 for Speedel, which led to promising and earlier-than-expected results from its renin inhibitor drug trials in hypertension. Progress was reported to be faster than classical development timeline, thanks to microdosing protocols (https://www.thepharmaletter.com/article/microdosing-boosts-progress-of-speedel-s-renin-inhibitor-trial, dated 21 February 2005), as also referenced by Robinson, 2008. In this case, three candidate compounds were compared, including SPP630 and SPP635, again both via i.v. and oral microdosing, and the compound demonstrating the highest oral bioavailability from the microdose study was selected for further development in Phase 2 clinical trials.

At least 10 other Phase 0 ^14^C-microdose studies were conducted in our company between 2006 and 2015, mostly using the designs as described above. The study results of these were not published, and these studies will not be discussed in detail here.

### 5.5 Phase 0 microdose candidate selection studies without ^14^C

Starting from 2015, we have occasionally seen Phase 0 microdose studies being conducted with drug material that was not labeled with ^14^C [or any other (radio-) isotope]. Such studies can be rather unremarkable in terms of designs or objectives, in that they may well aim to evaluate the microdose PK of candidate drugs in healthy male subjects. As an example, we conducted a study with three separate cohorts of six healthy male subjects aged 18–45 years, each receiving a single i.v. microdose of 100 μg; the main objective was to obtain early plasma PK data (including apparent t_½el_, CL, and V_d_) of single i.v. microdoses of the three molecules in humans. Non-clinical experiments in rats and dogs had indicated that all three compounds had excellent PK characteristics (and pharmacological activity), but the uncertainty in predicting human PK, in particular CL, from non-clinical species had been found to be a consistent problem with this specific drug class. Non-clinical toxicity testing comprised a single-dose i.v. toxicity study in rats with a 14-day post-dose observation period. Drugs were administered to the rats at a dose of 1.7 mg/kg, yielding a 1000-fold margin *versus* the proposed human dose of 100 µg. Moreover, clinical experience had already been obtained with drugs with the same mode of action, and no special risks were anticipated based on this clinical experience or non-clinical testing with the candidate drugs, as per ICH M3 R2 (2009). PK sampling was done for 72 h or 96 h after dosing depending on the candidate molecule. The quantification of drug concentrations in plasma was with a very sensitive LC-MS/MS method, lowest level of quantification of approximately 3 pg/mL for all three drug candidates.

Large PK differences were observed between the three candidate molecules, with a 4-fold range in Cmax, 59-fold range in AUC, 4-fold range in Vd, 67-fold range in CL (from low to very low), and 8-fold range in t1/2 (between 6 and 46 h). Interestingly, five subjects (28%) reported a total of six treatment-emergent adverse events. These included two cases of orthostatic hypotension (considered not related to the study drug) and one case of papular rash (considered possibly related to the study treatment by the investigator). The study was regarded useful to support further investigations on the molecules, although a preferred candidate was not immediately identified.

In another example, we conducted an i.v. Phase 0 comparative PK study to assess the properties of two molecules with the same mode of action, where one of them is the lead compound and the other is a back-up compound with (assumed) improved PK properties. The study was conducted in one cohort of five healthy male subjects aged 18–45 years, who received both drugs in a fixed-sequence cross-over design. Drugs were administered as a 15-min infusion, with a washout of 72 h between the two drug administrations. Bioanalysis was conducted by the sponsor, using LC-MS/MS.

The sponsor decided to employ a sentinel approach to ensure optimal safety, even though this is conceptually not needed in Phase 0 microdose studies. CL was found to be 17% lower for the back-up compound compared to the lead compound; Vd was three-fold higher for the back-up compound, and plasma elimination half-life was four-fold longer for the back-up compound.

Conducting the Phase 0 i.v. PK study for the lead compound was regarded as acceptable based on the non-clinical package developed and the exposures seen in the FIH study with oral dosing, while the back-up compound was qualified according to ICH M3 R2 (2009), Approach 1 (extended single dose toxicity testing at 1.67 mg/kg in rats, 1000-fold the human microdose).

### 5.6 A special case: assessing the gastrointestinal fate of an oral melanocortin-1 receptor agonist in humans without oral non-clinical toxicity testing

A special application of microdosing was employed by Palatin Technologies to assess the distribution of PL8177, a potent and selective melanocortin-1 (MC-1) receptor agonist developed as a gastrointestinal anti-inflammatory agent in inflammatory bowel disease (IBD), after the oral administration of a microdose of [^14^C]-PL8177 as an Eudragit^®^ polymer-encapsulated formulation in humans (Dodd et al., 2023). Polymer encapsulation was designed to protect PL8177 from degradation in the upper gastrointestinal (GI) tract and allow its release in the lower GI tract, where it would be expected to exert its effect. PL8177 was initially developed and tested in humans as a subcutaneous injection and found to be well-tolerated in single doses up to 5 mg and multiple doses up to 3 mg QD for 7 days before switching to oral intake and the local release of PL8177 in the lower GI tract in humans. The objectives of the study were to demonstrate the release of PL8177 from the polymer solid solution form of [^14^C]-PL8177 in the colon after oral administration through the observation of the main metabolite, PL8435; to confirm that the orally administered, free (released) [^14^C]-PL8177 did not result in systemic exposure to PL8177 and/or PL8435; and to establish the relationship between an oral dose of polymer formulated [^14^C]-PL8177 and the amount of free [^14^C]-PL8177 and its main metabolite in the colon. The decision to use a microdose approach allowed the oral study to be conducted without extensive oral toxicity testing. The study was conducted with a single dose of 70 µg of PL8177, labeled with 35 kBq (0.9 µCi) of ^14^C, in six cohorts of four healthy men aged 18–55 years each; in five of the six cohorts, a laxative was administered at a certain time after dosing. The laxative was administered at these different timepoints to ensure the excretion of the entire colon contents at different timepoints, which might allow for an assessment as to whether PL8177 was released from the polymeric oral colon release formulation inside the colon as expected. Free, ^14^C-labeled PL8177 and its main, pharmacologically active metabolite, ^14^C-PL8435, were detected in feces but not in plasma or urine in humans. Based on these data, it was concluded that PL8177 was released from the polymer formulation and metabolized within the GI tract, where it would be expected to exert its effect and that neither PL8177 nor PL8435 was absorbed into the systemic circulation. The data were regarded as supportive for further research into the oral formulation of PL8177 as a possible therapeutic for IBD in humans.

To support the human microdose study with the oral polymer product, a dedicated single-dose rat study was conducted with the maximum feasible dose of the polymer-bound drug product that could be administered orally as a single dose to rats, being 100 µg per rat. A safety margin of 70-fold was obtained *versus* the human 70-µg dose, based on BSA. Moreover, an assessment was made of the local exposure of the luminal surface of the GI tract after a 100-µg dose in rats *versus* the 70-µg dose in humans, and this was well above 1000-fold due to the much greater surface area in humans *versus* rats. These data, together with the available PK, safety, and tolerability data from the FIH study with s.c. dosing plus its supporting non-clinical package, were sufficient to run the human microdose study with the oral polymer-based dose of 70 µg of PL8177.

### 5.7 Another special case—microdose studies to assess drug–drug interactions

Several groups have published on the potential application of microdose studies in assessing drug–drug interactions (DDIs), with the potential that such approaches may be used in a very early stage of clinical drug development.

One of these investigations tested the effect of repeat dosing with combined pharmacological doses of ketoconazole and fluvoxamine (400 mg and 100 mg, respectively, inhibiting Pgp and CYP’s 1A2, 2C9, and 3A4) on a mixture of microdoses (“cassette microdosing”) of ^14^C-labeled midazolam (3A4 substrate), tolbutamide (2C9 substrate), caffeine (1A2 substrate), and fexofenadine (Pgp substrate) (25 μg and 1.85 kBq (50 nCi) each, with 100 μg and 7.4 kBq (200 nCi) total substrate microdoses per dosing occasion in total; Croft et al., 2012). The study was conducted in a cohort of six healthy male subjects aged 26–51 years; PK sampling was done for 72 h after the microdose mixture, and bioanalysis was done with LC + AMS in order to separate and subsequently quantify the ^14^C-labeled analytes. The study showed that AUCs increased by 13-fold for midazolam, 8-fold for caffeine, 3-fold for fexofenadine, and 2-fold for tolbutamide. These changes were consistent with those observed using the pharmacological doses of the probes and were taken to show that microdoses of potential DDI victims can be used to obtain an early indication as to the risk of investigational drugs being liable to drug–drug interactions. Linearity of PK parameters (all within 2-fold, mostly within 1.4-fold) between the microdose and pharmacological doses was observed for all probe drugs.

**FIGURE 3:**
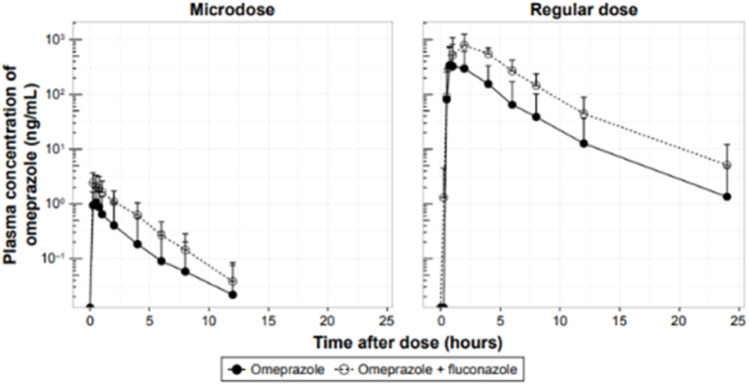
Effect of CYP2C19 inhibition on the PK of omeprazole administered as a 100-µg microdose or a 20-mg pharmacological dose in healthy male volunteers.

A similar example looked at omeprazole as the victim drug, administered as a 100-µg microdose and a 20-mg pharmacological dose, and the effect of CYP2C19 inhibition and induction with fluconazole and rifampin, respectively, on its PK (Park et al., 2017; Figure 3). The study was conducted in healthy male subjects aged 19–45 years, *n* = 6 per cohort. Bioanalysis was conducted using LC-MS/MS. The magnitude of increase in the AUC of the victim after fluconazole was 4.1-fold and 4.3-fold using the microdose and the pharmacological dose, respectively, and the AUC of the victim was reduced by 84% and 85% after rifampin administration using the microdose and the pharmacological dose, respectively. As mentioned above, the investigation concluded that microdose DDI studies may replace regular-dose studies or at least may be suitable for DDI screening purposes.

As a third and last example, investigators at Merck and Co. validated a microdose cocktail containing midazolam, dabigatran, pitavastatin, rosuvastatin, and atorvastatin to allow the simultaneous assessment of potential interactions at a selection of drug-metabolizing enzyme (CYP3A) and transporters (OATP1B, BCRP, and Pgp) (Prueksaritanont et al., 2017). The clinical utility of the microdose cocktail was demonstrated by conducting DDI studies with three DDI perpetrators (rifampin, itraconazole, and clarithromycin). As the outcome, the changes in the PK profiles of the probe substrates with the perpetrators were comparable to those seen after their respective pharmacological doses. As an exception, dabigatran showed an approximately 2-fold higher change in exposure with the microdose compared to the conventional dose, which was regarded as the microdose approach indicating a worst-case scenario for assessing the interaction potential of a victim drug at the Pgp level.

Finally, the authors of this analysis are aware of at least one microdose DDI study that was conducted as the second study in the clinical development program of a novel, investigational drug to assess CYP3A4 DDI liability using a validated 3A4 inhibitor as the perpetrator. The outcome of this study is pending publication.

### 5.8 A Phase 0 study to support the candidate selection of a long half-life drug

We recently completed a human microdose PK study, aiming to compare the PK of three structurally related, ^14^C-labeled candidate drugs in order to be able to assess whether one (or more) of the molecules would have the properties required to support further clinical development. The design was uncommon to the extent that PK sampling was to be conducted until 9 weeks after a single i.v. dose, which was considered necessary and useful because the drugs had been engineered to be long-acting. Moreover, the sponsor decided to employ sentinel dosing (one subject per cohort initially; no placebo treatment in the study, as usual for Phase 0 microdose studies) to mitigate risks to the maximum possible extent, even though this is conceptually not needed in Phase 0 microdose studies, especially also when the pharmacology of the active moiety is well-understood. PK in humans was assessed for the administered drug and for the active moiety, using LC + AMS. The non-clinical package comprised PK data in rats and monkeys, extended single-dose toxicity data in rats (three dose levels plus controls, between 0.16 and 1.5 mg/kg, in order to have a dosing option below 100 µg in case the highest dose would not be without observations), *in vitro* off-target screening, and *in silico* genotoxicity screening. No *in vivo* PD was done with drug candidates because pharmacological activity was not expected based on the molecular structure, and the pharmacological activity of the active moiety was well-characterized in humans. ^14^C-labeled API was manufactured under full GMP as per the sponsor’s policies. The amount of drug administered in the human microdose study was approximately 30 μg, based on PK simulations using existing knowledge about pharmacologically active doses in humans obtained from studies with the active moiety; the ^14^C dose was 10 kBq (270 nCi). The study was conducted in three parallel cohorts of six healthy male subjects each, aged 18–54 years, and all subjects completed the study as per the protocol except one case of SARS-CoV-2 infection. The outcome of the study was regarded as a success, showing different behaviors of the drug candidates in humans compared to rats and monkeys, and allowed the company to decide on further clinical development.

### 5.9 Other microdose/microtracer applications in the early clinical development

#### 5.9.1 Microdosing in FIH studies

Although strictly speaking not a Phase 0 approach, an interesting application of microdosing early in clinical development is the inclusion of an oral or i.v. micro (tracer) dose in FIH studies for orally administered drugs. The oral microtracer dose will be labeled with ^14^C, possibly as little as 3–4 kBq, ∼100 nCi, and serve to obtain early information on metabolism of the new molecule in humans (Lappin and Seymour, 2010), while the i.v. microdose will serve to assess absolute (oral) bioavailability in an early stage of clinical development, using either ^13^C or ^14^C labeling. As an alternative approach, one of the cohorts in the single ascending dose (SAD) phase of the FIH study may be selected to only receive an (unlabeled) i.v. microdose (i.e., without a concomitant oral dose) so as to obtain i.v. PK parameters (CL and Vd) early in clinical development (Young et al., 2023). Again, these are formally not Phase 0 studies since the (oral) doses to be investigated in these studies are targeted to reach the pharmacologically active range; however, these studies do allow important early clinical development objectives to be met with a microdose and, therefore, serve a purpose (and use technology) similar to Phase 0 studies. As an important advantage, such studies can be conducted without the need for human dosimetry calculations or GMP-quality ^14^C-labeled API, and without i.v. toxicity testing.

The above approach has been applied in a small number of published cases (Muehlan et al., 2018; in Young et al., 2023) and only once in our own institute (not published). As one potential disadvantage, we have found that the *a priori* decision in which (single dose) cohort of the FIH study the oral or i.v. microdose should be tested is often regarded as high risk; moreover, planning for and having the ^14^C-labeled drug material available and setting up the CMC work for the labeled material are perceived as potential hurdles. Evidently, these disadvantages do not apply when administering an (unlabeled) i.v. microdose as a stand-alone treatment in a dedicated cohort in the FIH study. We expect that the application of microdose/microtracer approaches in FIH studies will increase in the future as sponsors will increasingly adopt the regulatory opportunities that exist today.

#### 5.9.2 Microdosing combined with microdialysis or PET

An important field of research related to Phase 0 microdose studies attempts to relate systemic drug concentrations and PK parameters to tissue concentrations and tissue distribution. This is especially relevant since many drugs will exert their effects in tissue(s) rather than in blood or plasma. One approach that is currently being developed and employed on an increasing scale is intra-target (or intra-tissue) microdosing; this has been reviewed in Burt et al. (2017b). An earlier approach is to combine microdosing with imaging techniques, such as positron emission tomography.

In an early validation study, Wagner et al. (2011) assessed the plasma and brain PK of verapamil (used as a model compound labeled with either ^11^C or ^14^C) in six healthy male subjects. All subjects were administered an i.v. microdose (64 µg), followed by the same i.v. microdose concomitantly with an oral therapeutic dose (80 mg) in a two-way cross-over design. The i.v. dose was a mixture of 4.1 kBq (111 nCi) of ^14^C-verapamil and 411 MBq (11.1 mCi) of ^11^C-verapamil, and the oral dose was unlabeled verapamil. Brain exposure to ^11^C-radioactivity was measured with PET, whereas plasma exposure to (R)- and (S)-^14^C-verapamil was determined with LC-AMS. The study showed that the combination of AMS and PET microdosing allowed the characterization of plasma PK while at the same time providing data on verapamil brain PK.

Another potential approach to assess target-site (i.e., tissue) concentrations and tissue distribution of microdoses of investigational drugs is microdialysis. An interesting validation study was published recently, using ciprofloxacin as the model compound (Oesterreicher et al., 2022). In this study, nine healthy male subjects aged 18–55 years each received an i.v. injection of the ^14^C-labeled study drug (1.1 µg, 7 kBq/190 nCi) with or without an i.v. infusion of unlabeled ciprofloxacin at a therapeutic dose level (400 mg). Subcutaneous and intrapulmonary drug concentrations were assessed using microdialysis and bronchoalveolar lavage, respectively; the microdose was quantified using AMS, and the therapeutic dose using LC/MS-MS. Bronchoalveolar lavage was performed once in each subject; overall, three timepoints were assessed in three subjects each. The study found that the dose-normalized AUC of ^14^C-labeled ciprofloxacin in the subcutaneous tissue of the upper thigh was within 0.8–1.1-fold of the therapeutic dose exposure but that the exposure measurements of microdose ciprofloxacin in the lungs were variable and did not predict the epithelial lining fluid concentrations of therapeutic-dose ciprofloxacin well. Therefore, the potential application of microdoses to assess skin tissue concentrations appears feasible based on this study, but whether this also applies to lung tissue remains to be explored.

Interestingly, an older microdose PK study on an investigational antibiotic had suggested that the concentrations of the study drug in lung mucosa and alveolar macrophages after i.v. administration were clearly higher than those observed in the plasma (Lappin et al., 2013).

## 6 I.V. microdose ABA studies

Although technically speaking not microdose studies, and certainly not Phase 0 studies, in the strictest sense of the word, studies employing an i.v. ^14^C-labeled or occasionally ^13^C-labeled microdose to assess the ABA of a drug in humans are a very informative and popular application of microdoses in early clinical development. The fact that these are not strictly microdose studies arising from the fact that, in addition to the i.v. microdose, an oral (or, if applicable, another non-parenteral route of administration) dose in the pharmacological range is being administered.

The concept of i.v. ^14^C oral ABA studies builds on the combined administration of an unlabeled oral dose in the pharmacological dose range, which is then quantified in plasma using regular bioanalytical techniques (mostly LC-MS/MS), with a concomitant i.v. microdose that contains the same drug but labeled with ^14^C or ^13^C, and is ideally administered around Tmax of the oral dose. Plasma concentrations derived from the i.v. dose, representing the i.v. PK of the drug, will be assessed using AMS (for ^14^C labeling) or LC-MS/MS (for ^13^C labeling), allowing for AUC after i.v. dosing to be compared to the AUC from the oral dose, and, after dose-normalization, the calculation of ABA. As the two major advantages of this approach, the PK of the oral and the i.v. dose are being assessed in the same subject at the same point in time (each blood sample drawn will generate an “oral” and an “intravenous” drug concentration), thus preventing any complications arising from potential time-dependency. This is true because for the body, the molecules arising from the oral and the i.v. administration are indistinguishable. The other advantage with this approach is that for the i.v. microdose, no i.v. toxicity testing is required. The latter is based on the assumption that exposures after an i.v. microdose have been covered by prior non-clinical and especially clinical exposures (even if arising from oral administration), given the requirement that the microdose is to be no more than 1/100th of the expected PAD and has, therefore, been covered in the early escalation phase of the preceding FIH study.

The administration of the i.v. microdose around Tmax of the oral/non-parenteral dose will allow for the elimination of the labeled and unlabeled molecules to occur in the same timeframe, ensuring that the elimination of both the oral and i.v. doses will occur under identical physiological conditions.

As another advantage, similar to true Phase 0 microdose studies, the i.v. microdose ^14^C-labeled API does not need to be synthesized under GMP (Figure 2), although the i.v. drug formulation will need to be manufactured under cGMP. Moreover, in i.v. microdose ABA studies, there is a smaller risk of formulation issues with i.v. solution since the concentration/amount of drug is very low compared to traditional two-way cross-over oral vs. i.v. ABA studies. At the same time, the low concentrations of the drug in the i.v. solution may show increased liability when it comes to drug adsorption to materials since adsorption is generally saturable and, therefore, represents a higher percentage of a lower magnitude dose. It is strongly advised to always assess this risk of drug adsorption of microtracer drug amounts during the manufacture and analysis of the mock or technical formulation. The formulation can either be an i.v. injection or an i.v. infusion; infusions are preferred because they allow for a more accurate estimate of the plasma concentration at t = 0, C_0_, and come with a lower risk of tolerability issues.

The data obtained from the i.v. microdose study (CL and Vd, in addition to ABA) will allow a further and better understanding of the drug’s properties, will help in building an accurate PBPK model, and will help assess the risk that the drug may suffer from low or variable absorption, changes in absorption with food, and changes in PK due to drug–drug interactions. ABA, as assessed using i.v. microdose designs, is accepted for market approval by FDA, EMA, Health Japan, and TGA (Australia). An interesting option is to run i.v. microdose ABA after a single dose of the investigational drug and then repeat it after repeat dosing, in order to assess whether changes in CL, Vd, or F have occurred with repeat dosing.

The sample size for this type of study is not regulated, and we have seen numbers as small as 4 or as large as 12 subjects, the latter being typically healthy volunteers but occasionally cancer patients. The sample size is mainly driven by study objectives in conjunction with (expected) PK variability and cost.

An intrinsic aspect of i.v. microdose ABA studies is that the molecules arising from the oral and the i.v. administration should be distinguishable when analyzing plasma concentrations. In the vast majority of cases, this is enabled by labeling the i.v. dose with ^14^C, but in some cases, this is done by labeling the i.v. dose with ^13^C. Using ^13^C for labeling purposes has the advantage of not using a radioisotope, with an associated convenience of avoiding radiosynthesis, need for certified pharmacy and clinical unit, and need for AMS. At the same time, the chemistry of the investigational compound should allow the introduction of a sufficient number of ^13^C atoms into the molecule, in order to generate sufficient bioanalytical sensitivity on top of the natural ^13^C abundance of 1.1%, as opposed to natural ^14^C abundance being 1 part per trillion. The bioanalysis of ^13^C-labeled drug is done with LC-MS/MS.

As the most efficient study design, assuming one will be capable of producing the ^13^C-labeled drug material with sufficient labeling, one may decide to administer the ^14^C-labeled investigational drug orally and the ^13^C-labeled microdose intravenously, around the Tmax of the oral dose. In this manner, ADME and ABA objectives can be met in one cohort of study subjects and in one sample collection period. The oral ^14^C dose may be a microtracer dose or a “regular” dose (generally defined as 3.7 MBq, or 100 µCi), whilst the i.v. dose will be a microdose.

An excellent review covering i.v. microdosing in drug development has been published recently (Young et al., 2023). For selected examples of i.v. microdose studies to assess ABA, the reader is referred to a number of published papers (Graham et al., 2012; Hoffmann et al., 2012; Boulton et al., 2013; Raje et al., 2018; Schueller et al., 2022; Ma et al., 2023). Examples of a combined ^13^C/^14^C study to assess ABA and ADME are found in Schwab et al. (2013) and Guerini et al. (2017).

IV microdose ABA studies have also been conducted in cancer patients, for drugs developed for oncology indications and for which testing in healthy volunteers was seen as not acceptable. Examples can be found in Denton et al., 2013; Leonowens et al., 2014; Von Richter et al., 2016; Johne et al., 2020; Zhang et al., 2020. In one study, the combined ^14^C/^13^C approach for assessing ADME and ABA properties was employed in cancer patients as well (Pápai et al., 2019).

Finally, i.v. microdosing has also been employed for inhaled drugs as being an alternative non-parenteral route of drug administration (Harrell et al., 2019).

## 7 Future perspectives

A number of excellent reviews have been published which explored the concepts and future use and application of Phase 0 and microdose studies, and we kindly refer the reader to these (Dueker et al., 2011; Lappin et al., 2013; Burt et al., 2016; Burt et al., 2017a; Burt et al., 2018; Burt et al., 2020; Burt et al., 2022). However, as stated in the introduction to the current analysis, we will offer our views as to what appears to be needed to further increase the adoption of informative applications of Phase 0/microdose studies in the near future.

**FIGURE 4:**
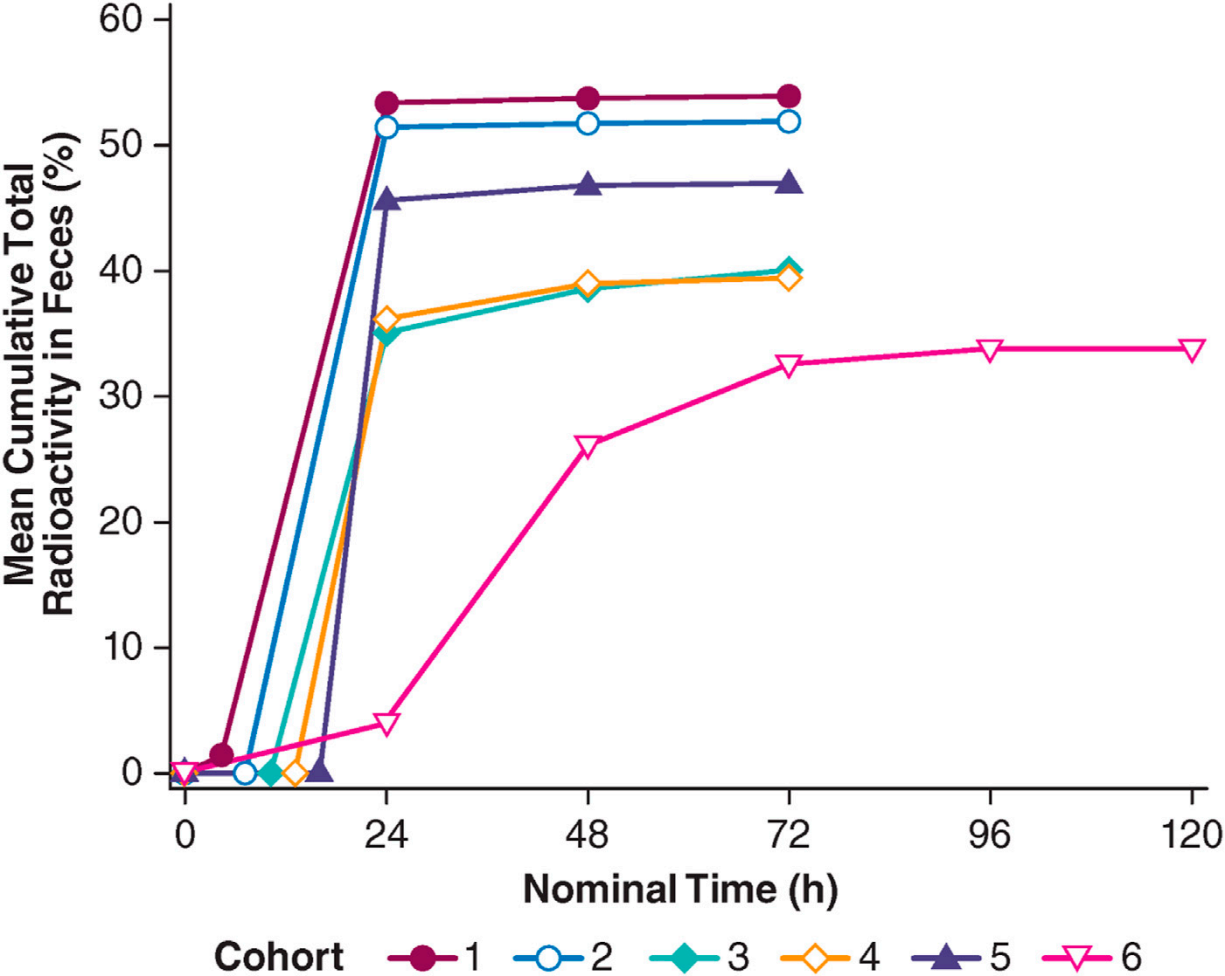
Total radioactivity excreted in feces after a single oral, polymer-encapsulated microdose of the melanocortin-1 receptor agonist, PL8177, in healthy male volunteers. Cohorts 1, 2, 3, 4, and 5: [^14^C]-PL8177 plus a laxative at 5, 8, 11, 14, and 17 h post-dose, respectively; cohort 6: [^14^C]-PL8177 without a laxative.

1. First and foremost, in terms of mindset, it will help us as an industry if we adopt Phase 0 studies as a means to learn early about and better understand the molecule(s) that we are developing, rather than seeing Phase 0 as a distraction that costs money and takes time.
2. Phase 0/microdose/microtracer studies should be implemented in drug development programs based on a need. In other words, in all cases in which human PK or i.v. PK parameters in humans are considered to be of value, such studies and approaches should be considered when planning for clinical development. Some will argue that such needs will always exist since early PK in humans will always trump PK in animal species, and Phase 0 approaches offer the testing of compounds at sub-therapeutic doses in humans, supported by a reduced pre-clinical package to enable early decisions and limit animal use. However, in our minds, each development program should consider the pros and cons of implementing Phase 0/microdose approaches in each specific case; the outcome of such considerations will come with a level of development risk reduction that has to be balanced against investments.
3. One of the major (perceived) hurdles to make the most use of microdose studies is the need to work with ^14^C-labeled drug material of GMP quality and analyze this in biological matrices with AMS. In our experience, and based on our benefit-risk approach (Figure 4), adequately qualified but non-GMP ^14^C-labeled drug material can be acceptable for use in microdose studies and support safe and informative investigational drug studies. As an alternative, further efforts may be invested in developing LC-MS/MS methodology of sufficient sensitivity that will allow measuring drug concentrations arising from microdoses in humans without the use of ^14^C. For microdose PK studies with ^14^C-labeled drug material, an increase in the worldwide capacity in AMS or further development of novel technologies will help increase the adoption of microdose PK studies further.
4. From a regulatory perspective, there are no apparent hurdles when it comes to running Phase 0 microdose studies, especially none in the field of microdose PK studies. Therefore, it is up to us as an industry to understand and adopt the potential applications of Phase 0 microdose studies and associated designs, and develop new drugs to the best of our abilities.

## Abbreviations

ABA, absolute bioavailability; AMS, accelerator mass spectrometry; API, active pharmaceutical ingredient; AUC, area under the concentration *versus* time curve; Bq, becquerel; CHMP, Committee for Medicinal Products for Human Use; Ci, Curie; CMC, chemistry, manufacturing, and controls; CL, clearance; CREAM, Consortium for Resourcing and Evaluating AMS Microdosing; DDI, drug–drug interaction; EMA, European Medicines Agency; EUMAPP, European Union Microdose AMS Partnership Programme; FDA, (United States) Food and Drug Administration; FIH, first-in-human; GI, gastrointestinal; GMP, good manufacturing practice; IBD, irritable bowel disease; ICH, International Council for Harmonization (of technical requirements for pharmaceuticals and human use); ICRP, International Commission on Radiological Protection; IND, Investigational New Drug; LC-MS/MS, liquid chromatography with tandem mass spectrometry; PD, pharmacodynamic(s); PK, pharmacokinetic(s); SAD, single ascending dose; Vd, volume of distribution.
